# Optimized Analysis of Cervical Vertebrae Maturation in Cone-Beam Computed Tomography: A Cross-Sectional Study

**DOI:** 10.34172/joddd.42445

**Published:** 2026-03-30

**Authors:** Bárbara Pilla Tavares, Kelly Galisteu-Luiz, Luciana Duarte Caldas, Luciano Santos Pinto Guimarães, Lincoln Issamu Nojima, Maria Augusta Visconti, Matilde da Cunha Gonçalves Nojima

**Affiliations:** ^1^Department of Pediatric Dentistry and Orthodontics, Universidade Federal do Rio de Janeiro, Rio de Janeiro, RJ, Brazil; ^2^Guimarães LSP Consultoria Científica Ltda, Florianópolis, SC, Brazil; ^3^Department of Pathology and Oral Diagnosis, Universidade Federal do Rio de Janeiro, Rio de Janeiro, RJ, Brazil

**Keywords:** Age determination by skeleton, Cervical vertebrae, Cone-beam computed tomography, Growth and development, Orthodontics

## Abstract

**Introduction:**

Cone-beam computed tomography (CBCT) scans are widely used in dentistry as a valuable and accurate research tool for assessing skeletal age. This study aimed to conduct an optimized analysis of cervical vertebrae maturation using pre-existing CBCT images and compare the findings with mandibular growth stages.

**Methods:**

Seventy-five participants evaluated 24 CBCT images, from which 12 lateral cephalometric projections (Cef2D) and 12 sagittal tomographic reconstructions (Rec2D) were extracted. The inferior borders of the C2, C3, and C4 vertebrae, as well as the shapes of C3 and C4, were analyzed according to the cervical vertebral maturation (CVM) method proposed by Baccetti et al. (2005). The degree of reproducibility was assessed using Cohen’s kappa coefficient.

**Results:**

The analysis of intra- and inter-examiner reproducibility for the inferior borders of the cervical vertebrae and the classification of maturation stages showed almost perfect and substantial levels of agreement, respectively. These results were consistent for both Cef2D and Rec2D images. When mandibular growth stages were grouped, the reproducibility of the CVM method also reached an almost perfect level of agreement for both image types.

**Conclusion:**

The analysis of CVM, adapted from the method by Baccetti et al. (2005) for CBCT, demonstrated a high level of reproducibility, particularly when evaluating the inferior borders of the C2, C3, and C4 vertebrae and when grouping post-peak stages of mandibular growth.

## Introduction

 The analysis of skeletal maturation, represented by the morphological assessment of bone mineralization stages, is highly relevant for diagnosis and orthodontic planning, as it demonstrates a high level of scientific reliability compared to chronological age analysis.^1,2^ Traditionally, several methods for predicting skeletal maturation have been proposed based on two-dimensional (2D) imaging data, such as the evaluation of calcification stages in the carpal region on hand and wrist radiographs,^1,3^ and the assessment of cervical vertebrae maturation (CVM) on lateral cephalometric radiographs.^4-6^

 Controversies in the literature regarding the use of three-dimensional (3D) imaging for cervical vertebrae analysis underscore the importance of this study. While some research highlights the method’s high reproducibility,^4,6-15^ others report inconsistencies, suggesting that it should not be used as the sole diagnostic guideline.^16-21^

 Given the rapid advancements in radiology and diagnostic technology, 3D studies evaluating skeletal maturation have shown significant growth.^8,22-26^ Therefore, this study aimed to estimate skeletal age through an optimized analysis of cervical vertebrae maturation (CVM) in pre-existing cone-beam computed tomography (CBCT) images and compare the findings with mandibular growth stages. The objective was to address unresolved questions regarding cervical vertebrae skeletal maturation while minimizing additional radiation exposure solely for research purposes.

## Methods

###  Procedural Steps

 This study was approved by the Human Research Ethics Committee at the Clementino Fraga Filho Hospital, Universidade Federal do Rio de Janeiro (CAAE 71244217.9.0000.5257, opinion number 2.203.180/2017). All the participants who agreed to take part in the study received an informed consent form at the beginning, which was read in its entirety and signed.

###  Sample Size

 A sample size calculation was performed using the WinPEPI software, version 11.65 (Brixton Health, London, UK),^27^ applying Cohen’s kappa agreement test. A significance level of 5% (α = 0.05), an agreement frequency of 50%, and a kappa coefficient of 0.8 were set to determine the minimum sample size needed to ensure statistical robustness. Based on the lower bound of the 95% confidence interval, set at 0.75, the ideal sample was determined to consist of 474 responses. Therefore, considering the need to assess the stages of cervical vertebrae maturation, a minimum of 12 CBCT scans (two different scans to represent each of the six cervical vertebrae maturation stages) and 40 participants were required, totaling 480 responses.

###  Obtaining and Selecting Imaging Exams

 Twelve pre-existing CBCT scans from patients who began treatment at the Orthodontics Clinic of the Postgraduate Program in Dentistry at the Universidade Federal do Rio de Janeiro were evaluated. Based on the established inclusion criteria, the selected CBCT scans were from individuals of both sexes, aged between 7 and 20 years, whose images clearly depicted the C2, C3, and C4 cervical vertebrae. CBCT scans were excluded if they were from individuals with syndromes or any anomalies in the growth and/or development of the craniofacial complex, as well as those presenting morphological changes in the cervical vertebrae.

 All the examinations used were obtained with a cone-beam tomograph (Kodak 9500^®^ Cone Beam 3D System; Carestream Health, Rochester, NY, USA), following an acquisition protocol with a fixed FOV of 18.4 × 20.6 cm and automatic adjustment of the other parameters—90 kVp, 10 mA, isotropic voxel size of 0.3 mm³, and a scanning time of 24 seconds. The examinations were conducted with patients in a corrected posture, with a straight back and head at rest, maintaining the Frankfort plane parallel to the floor.

 The examination results were exported as Digital Imaging and Communications in Medicine (DICOM) files and analyzed using Dolphin Imaging^®^ software, version 11.7 Premium (Dolphin Imaging & Management Solutions, Chatsworth, California, USA). The head position was standardized according to the reference planes (axial, coronal, and sagittal).^28^

 High-quality lateral cephalometric radiographs (Cef2D) and two-dimensional images of the cervical vertebrae (Rec2D) were generated from the most central sagittal reconstructions of the CBCT, ensuring complete coverage of these structures.^26^ In the Cef2D projections, images of the spheno-occipital synchondrosis and dental arches were excluded to maintain focus on the C2, C3, and C4 cervical vertebrae, ensuring that these structures did not interfere with the maturation analysis. The Rec2D images were generated using the midline as a reference to exclude the processes of the cervical vertebrae and adjacent anatomical structures. For each patient, the selected sagittal reconstruction provided the clearest representation of the entire vertebrae of interest, with slice thickness adjusted as needed.

 From the 12 CBCT exams selected, 24 images—comprising 12 Cef2D and 12 Rec2D—were obtained to perform a qualitative assessment of CVM, following the method described by Baccetti et al.^6^

###  Image Analysis

 Four examiners experienced in the CVM method—two orthodontists and two radiologists—performed a preliminary analysis. Following intra- and inter-examiner calibration, a kappa coefficient indicating 100% agreement in responses among the four examiners was obtained.

 The C2, C3, and C4 cervical vertebrae were evaluated through visual inspection of their morphological features, specifically assessing the presence or absence of concavity on the inferior borders of their bodies and their shapes (trapezoidal, horizontal rectangular, quadrangular, or vertical rectangular). Subsequently, the vertebral maturation stages were classified according to the CVM method.^6^

**Figure 1:**
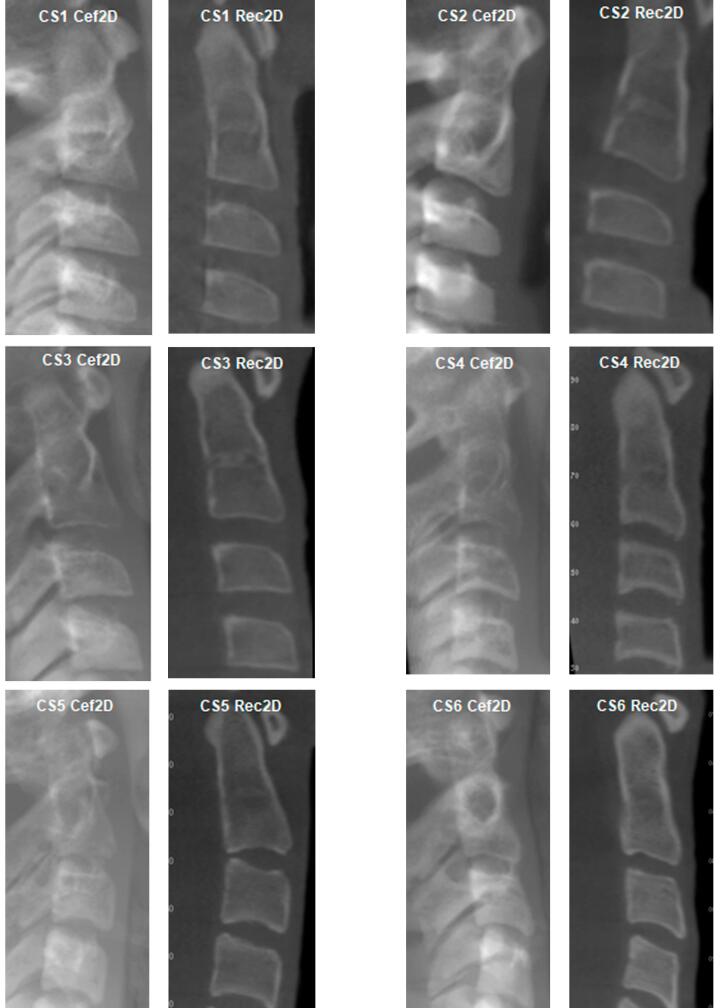
Two-dimensional Cef2D and Rec2D images representing cervical vertebrae maturation stages (CS1–CS6) according to the CVM method proposed by Baccetti et al. (2005)

 Once the Cef2D and Rec2D images had been obtained, professors and postgraduate students in Orthodontics were invited to participate in evaluating the reproducibility of the CVM method. Each of the 75 participants evaluated 24 images (12 Cef2D and 12 Rec2D), totaling 1,800 responses at each time interval of the study. Figure 1 illustrates the Cef2D and Rec2D images considered in this study, representing each CVM stage according to the classification described by Baccetti et al.^6^

 For each image, all the participants completed evaluation forms, answering the following questions regarding the morphology of the C2, C3, and C4 vertebrae: 1) whether the inferior borders of C2, C3, and C4 were straight or concave; 2) whether the shapes of the C3 and C4 cervical vertebrae were trapezoidal, horizontal rectangular, quadrangular, or vertical rectangular; 3) whether the stage of maturation of the cervical vertebrae would be classified as CS1, CS2, CS3, CS4, CS5, or CS6.

 Before the first evaluation period (T1), a theoretical presentation was provided in the form of a video explaining the CVM method and offering instructions for completing the evaluation forms. To support participants during the analyses, a flyer was distributed containing graphic representations of the cervical vertebrae maturation stages.^6^

 All the images were presented for evaluation in random order and projected individually. Each participant answered the aforementioned questions regarding the morphology and maturation stage of the cervical vertebrae for both Cef2D and Rec2D images.

 Four weeks after T1, the second evaluation period (T2) was conducted. Under similar conditions, all the participants watched the instructional video on the CVM method again and repeated the process of analyzing the images presented during T1. The images were randomized again before T2, resulting in a total of 3,600 responses for the study (T1 + T2).

 In investigating the parameters used as guidelines in the CVM method, this study aimed to answer the following questions: What is the degree of reproducibility in visualizing concavity on the inferior border of the cervical vertebrae? What is the degree of reproducibility in evaluating the shape of the body of the cervical vertebrae? Regarding the classification of vertebral maturation stages proposed by Baccetti et al.,^6^ what is the degree of reproducibility of this method? Since orthopedic treatments are performed before and during the pubertal growth spurt,^6,16,17^ what is the degree of reproducibility of the CVM method when considering the grouping of post-peak mandibular growth stages?

###  Statistical Analysis

 The variables analyzed were represented by absolute and relative frequencies. Cohen’s kappa coefficient was used to assess intra- and inter-examiner reproducibility, based on the following agreement parameters:

 0: poor

 0.01–0.20: weak

 0.21–0.40: reasonable

 0.41–0.60: moderate

 0.61–0.80: substantial

 0.81–1.00: almost perfect^29^

 Statistical analysis was performed using SPSS 18.0 (SPSS Inc., Chicago, IL, USA), with a significance level set at 5% (*P* ≤ 0.05).

## Results

 Analysis of the data revealed divergences in the responses regarding the inferior borders of the C2, C3, and C4 vertebrae, the shape of the bodies of the C3 and C4 vertebrae, and their corresponding maturation stages.

 The response combinations were categorized as either coherent or incoherent (Table 1). A coherent analysis was defined as a combination of responses for the inferior border and vertebral shape that matched the stages predicted by the CVM classification.^6^ This group comprised 2,718 responses across both time intervals (T1 and T2), representing 75.5% of the sample. An incoherent analysis arose from two situations: (I) when the combination of responses regarding the inferior border and vertebral shape did not correspond to any valid stage according to the CVM method (invalid stage); or (II) when the combination led to a valid stage, but the examiner incorrectly classified it (classification error). In total, 882 incoherent responses were identified across T1 and T2, representing 24.5% of the sample.

**Table 1:** Coherences and incoherences identified in the evaluation of two-dimensional Cef2D and Rec2D images during T1 and T2 evaluation periods

			**Cef2D**		**Rec2D**	
			**n**	**%**	**n**	**%**
T1	Coherent		704	78.2	623	69.2
	Incoherent	Invalid stage	115	12.8	170	18.9
		Classification error	81	9	107	11.9
T2	Coherent		710	78.9	681	75.7
	Incoherent	Invalid stage	116	12.9	149	16.6
		Classification error	74	8.2	70	7.8

n Cef2D = 900, n Rec2D = 900, for T1; n Cef2D = 900, n Rec2D = 900, for T2

 For analyses concerning the inferior borders of C2, C3, and C4, and the shapes of C3 and C4, all the responses were considered. Regarding vertebral maturation stages based on the CVM method,^6^ three assessment models were established: (I) General CVM Stage—all responses were considered; (II) Coherent CVM Stage—only responses without inconsistencies; (III) Grouped Coherent CVM Stage—consistent responses in which post-mandibular growth peak stages were grouped.

Tables 2 and 3 present inter- and intra-examiner agreement levels, as expressed by Cohen’s kappa coefficient, respectively.^29^

**Table 2:** Inter-examiner agreement for the variables used in the analysis of cervical vertebral maturation in two-dimensional Cef2D and Rec2D images during T1 evaluation period of the study

		Inferior border**	Shape**	General CVM stage**	Coherent CVM stage***	Grouped coherent CVM stage***
Cef2D	Agreement n (%)	798 (88.7)	527 (58.6)	632 (70.2)	461 (76.3)	555 (91.9)
	Kappa (p)*	0.83	0.454	0.643	0.714	0.882
	Agreement level	Almost perfect	Moderate	Substantial	Substantial	Almost perfect
Rec2D	Agreement n (%)	810 (90.0)	490 (54.4)	688 (76.4)	460 (86.6)	509 (95.9)
	Kappa (p)*	0.85	0.441	0.718	0.836	0.927
	Agreement level	Almost perfect	Moderate	Substantial	Almost perfect	Almost perfect

*Kappa *P* < 0.001; **n Cef2D = 900, n Rec2D = 900; ***n Cef2D = 604, n Rec2D = 531

**Table 3:** Intra-examiner agreement for the variables used in the analysis of cervical vertebral maturation in two-dimensional Cef2D and Rec2D images

		Inferior border**	Shape**	General CVM stage**	Coherent CVM stage***	Grouped coherent CVM stage***
Cef2D	Agreement n (%)	775 (86.1)	525 (58,3)	612 (68.0)	464 (76.8)	533 (88.2)
	Kappa (p)*	0.791	0.473	0.616	0.72	0.829
	Agreement level	Substantial	Moderate	Substantial	Substantial	Almost perfect
Rec2D	Agreement n (%)	785 (87.2)	533 (59.2)	621 (69.1)	443 (83.4)	496 (93.4)
	Kappa (p)*	0.805	0.499	0.626	0.796	0.881
	Agreement level	Substantial	Moderate	Substantial	Substantial	Almost perfect

*Kappa *P* < 0.001; **n Cef2D = 900, n Rec2D = 900; ***n Cef2D = 604, n Rec2D = 531

## Discussion

 The use of three-dimensional (3D) technology has revolutionized diagnostic imaging methods in orthodontics;^30^ however, the assessment of skeletal development stages through the analysis of CVM in CBCT scans has not been adequately explored. Recent studies have shown that CBCT scans can be used as a valid and reliable diagnostic tool for this purpose, comparable to cephalometric radiographs.^31,32^ Field of view (FOV) options meet the requirements for CBCT examinations in pediatric orthodontic patients with various specific indications, including 3D cephalometric analyses.^33^ Customized optimization strategies can provide more accurate and effective treatment planning, achieving reduced radiation doses through improved accessibility and minimizing the need for additional 2D images.^33‒35^ In this context, the ALADAIP principle (“as low as diagnostically acceptable, being indication-oriented and patient-specific”) plays a crucial role in minimizing radiation exposure, as comprehensive imaging of the entire head is essential for orthodontic planning.^36^

 The literature has reported controversies regarding the analysis of the morphological characteristics of cervical vertebrae, with studies revealing high reproducibility of the CVM method^4,7,10,13,14^ and levels of agreement ranging from almost perfect^6,8,9,11,12,19^ to substantial,^15^ while other studies reported low reproducibility.^16-18,20,21^ In the majority of previous studies, the degree of reproducibility of the CVM method evaluated exclusively the stage of maturation.^9,11,15,16,19-21,26,37,38^ However, it is important to consider the inclusion of morphological characteristics of cervical vertebrae and the classification of their stages, as performed by Nestman et al.^17^ and Sohrabi et al.^18^ Considering the inter-examiner assessment at T1 in the present study, the degree of reproducibility related to the inferior border of the vertebrae presented an almost perfect level of agreement (Table 2, Additional File 2), disagreeing with the moderate to substantial levels of agreement previously mentioned in the literature.^17,18^ However, regarding the shape, the level of agreement was moderate, which corroborates the findings of Sohrabi et al.,^18^ but differs from the reasonable level reported by Nestman et al^17^ (Table 2, Additional File 2). Regarding the intra-examiner assessment, the levels of agreement for the inferior border and the shape of the cervical vertebrae were substantial and moderate, respectively (Table 3, Additional File 3). In contrast, Sohrabi et al.^18^ found substantial to almost perfect levels of agreement for the inferior border and substantial levels of agreement for the shape of the cervical vertebrae.

 To date, this is the first study in which participants themselves directly determined the CVM stage based on their evaluations of the inferior borders and shapes of the cervical vertebrae. When the inferior border and vertebral shape did not clearly correspond to a stage defined in the CVM method, the participants tended to rely more heavily on the inferior border when making their final determination of the maturation stage. This is a noteworthy distinction, as in the studies by Nestman et al.^17^ and Sohrabi et al.,^18^ the CVM stage was not directly defined by participants but was determined by the authors based on participants’ assessments of the inferior border and vertebral shape.

 Out of the 3,600 responses in this study, 24.5% were classified as incoherent (Table 1, Additional File 1). A common error involved misclassifying responses that should correspond to stage CS5 as CS4. Similar rates of incoherent responses in CVM classification have been reported in the literature,^17,21,39^ ranging from 14% to 30%.^17,39^ In particular, frequent incoherencies have been noted in stage CS4,^39^ as well as the observation of horizontal rectangular shapes for C3 and C4 in some adult patients.^21^

 Concerning the degree of reproducibility relative to the Coherent CVM Stage, the level of inter-examiner agreement was almost perfect for Rec2D and substantial for Cef2D (Table 2, Additional File 2). This was the only difference observed between the two image types concerning the reproducibility parameters evaluated. These findings demonstrate a higher degree of reproducibility than those reported in previous studies, which indicated moderate inter-examiner agreement.^17,18^

 When comparing responses related to the inferior border between Cef2D and Rec2D for the same individuals, virtually no difference was observed in the present study. However, there was greater variability in shape-related combinations. Hassel and Farman reported that this could be associated with the presence of overlaps or noise in radiographic images, which reduces image clarity.^4^ The literature has consistently emphasized the importance of analyzing the inferior borders of the cervical vertebrae in relation to their maturation stages. San Román et al.^40^ evaluated the anatomical parameters of the inferior border, vertebral height, and shape, and verified that, when each of these parameters was individually compared with skeletal maturation based on hand and wrist radiographs, the concavity of the inferior border demonstrated the strongest correlation.

**Figure 2:**
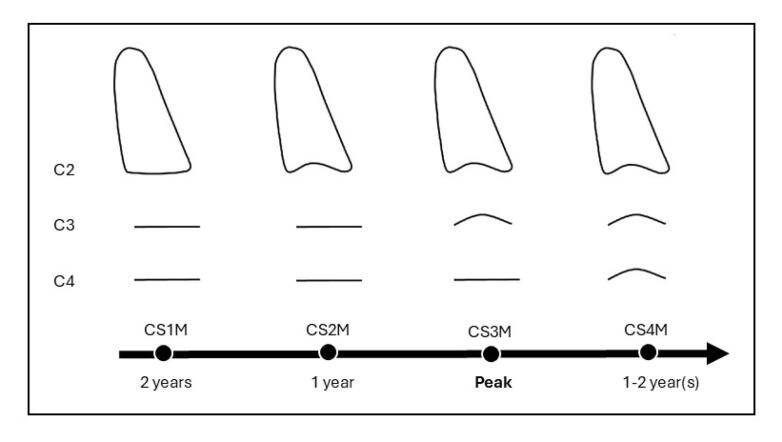
Diagram of the CVM method recommended by Baccetti et al. (2005), illustrating the inferior borders of vertebrae C3 and C4. Modified cervical stage 1 (CS1M): The inferior borders of C2, C3, and C4 are flat. The mandibular growth peak will occur, on average, two years after this stage. Modified cervical stage 2 (CS2M): The inferior border of C2 is concave, while those of C3 and C4 remain flat. The mandibular growth peak will occur, on average, one year after this stage. Modified cervical stage 3 (CS3M): The inferior borders of C2 and C3 are concave, and that of C4 remains flat. The mandibular growth peak will occur during the year in which this stage is identified. Modified cervical stage 4 (CS4M): The inferior borders of C2, C3, and C4 are all concave. The mandibular growth peak occurred at least one to two years before this stage

 Clinically, in cases of sagittal and transverse maxillary deficiency, the most appropriate time for orthopedic intervention is during the pre-pubertal phase, specifically at stages CS1 and CS2. For cases involving mandibular ramus deficiency and mandibular retrognathism, the ideal time for achieving more effective orthopedic treatment corresponds to the peak growth period, represented by stage CS3.^6,12,41,42^ In the literature, stage CS4 is described as a pubertal stage.^37,42^ However, it is important to note that, according to Baccetti et al.,^6^ the mandibular growth peak occurs at least one to two years prior to this stage. Based on the results presented, although the analysis of the inferior borders of the cervical vertebrae demonstrated an almost perfect level of agreement, the evaluation of vertebral shape remains a controversial factor that may compromise the reproducibility of the CVM method. Therefore, it is suggested that only the presence or absence of concavity on the inferior borders of the C2, C3, and C4 vertebrae should be considered, while grouping the post-peak mandibular growth stages—CS4, CS5, and CS6—since changes occurring in these stages are primarily related to vertebral shape (Figure 2).

## Conclusion

 The optimization of the cervical vertebral maturation (CVM) method proposed by Baccetti et al. (2005) for application in CBCT demonstrated a higher level of reproducibility, particularly when considering the inferior borders of the C2, C3, and C4 vertebrae, as well as the grouping of post-peak stages of mandibular growth.

## Competing Interests

 The authors declare no competing interests related to this research.

## Ethical Approval

 This study was approved by the Human Research Ethics Committee of the Clementino Fraga Filho Hospital, Universidade Federal do Rio de Janeiro, Brazil (CAAE 71244217.9.0000.5257, opinion number 2.203.180/2017). All the participants who agreed to take part in the study gave their informed consent before starting it.
